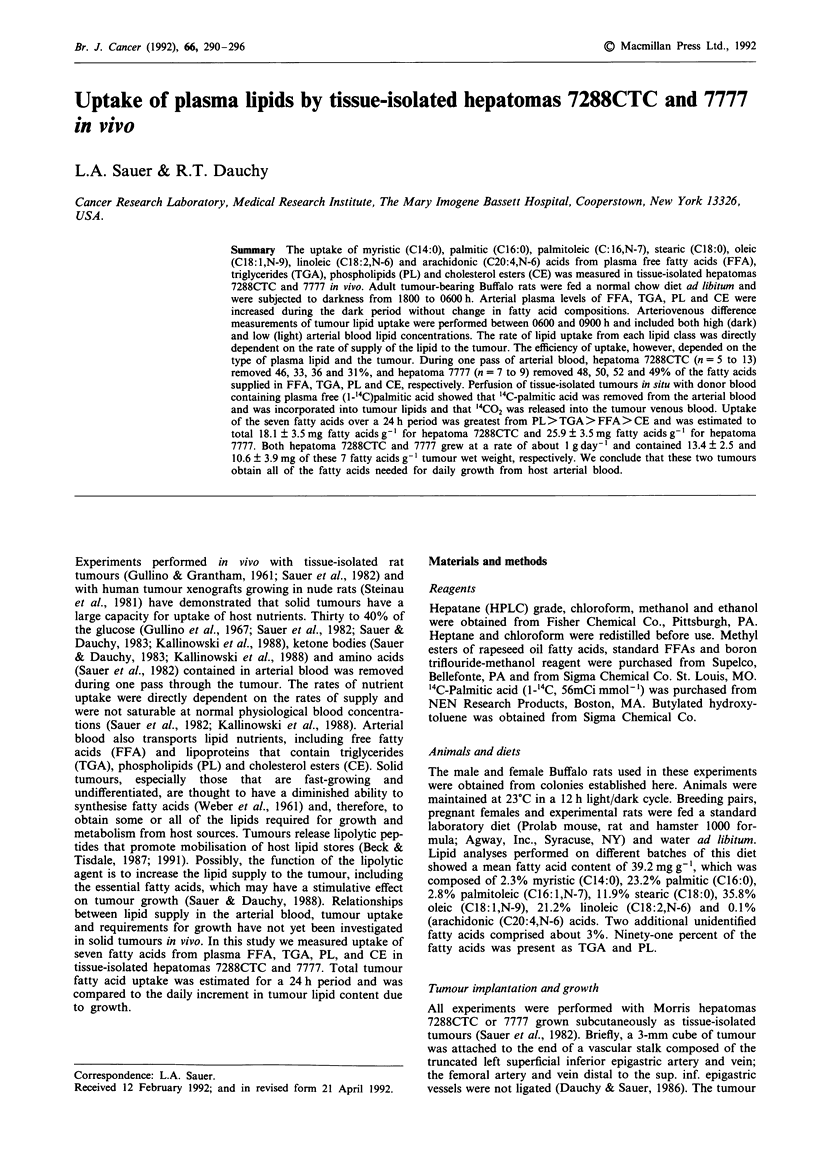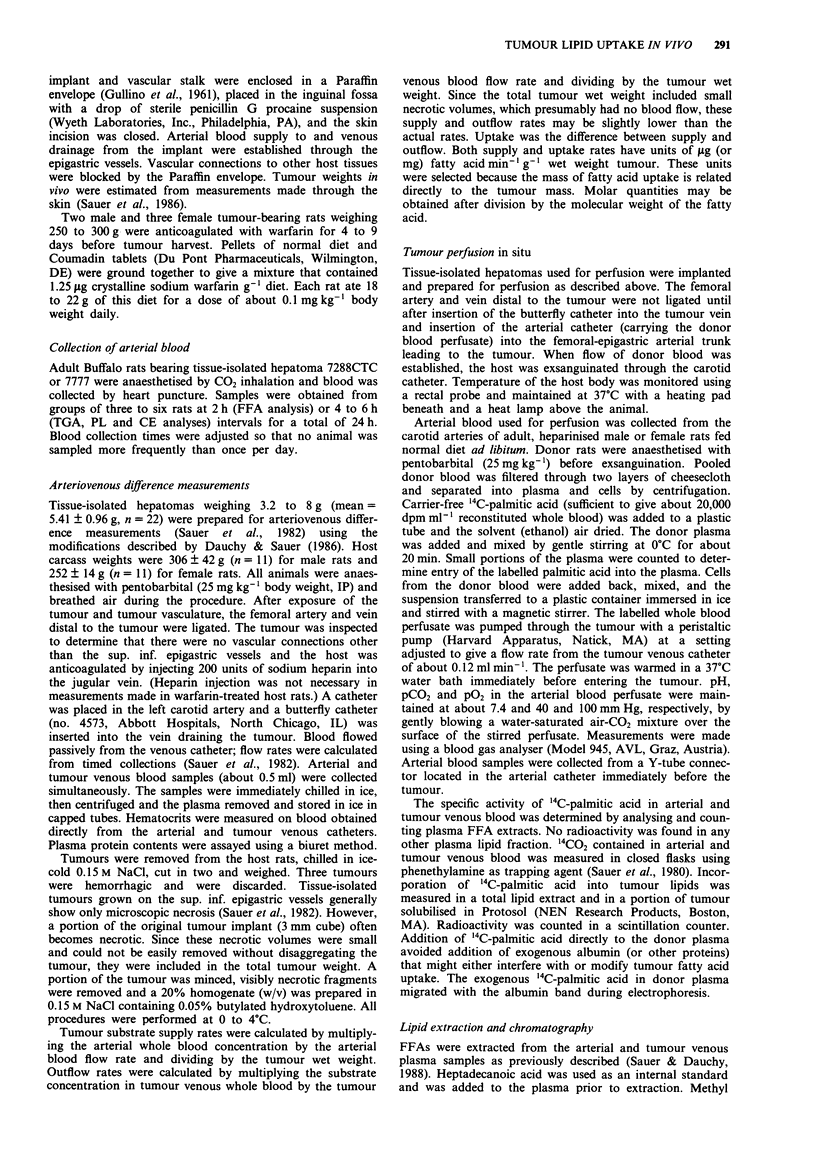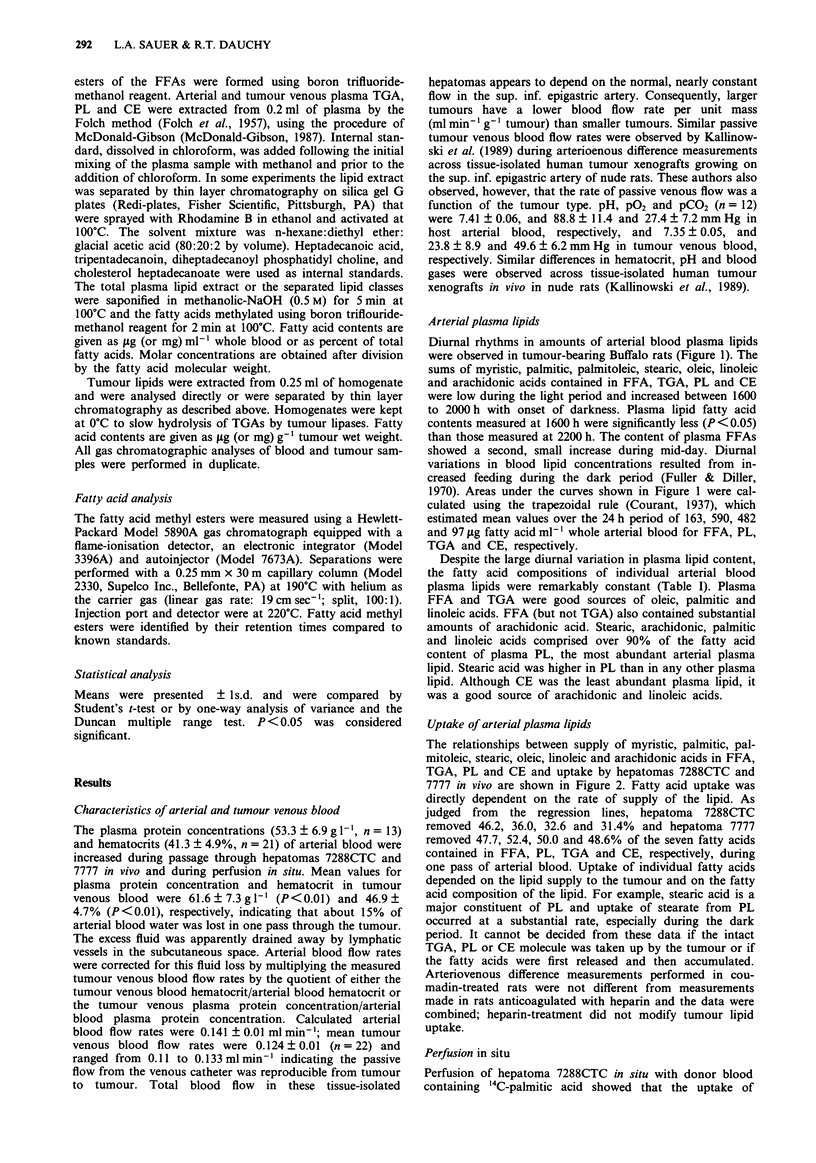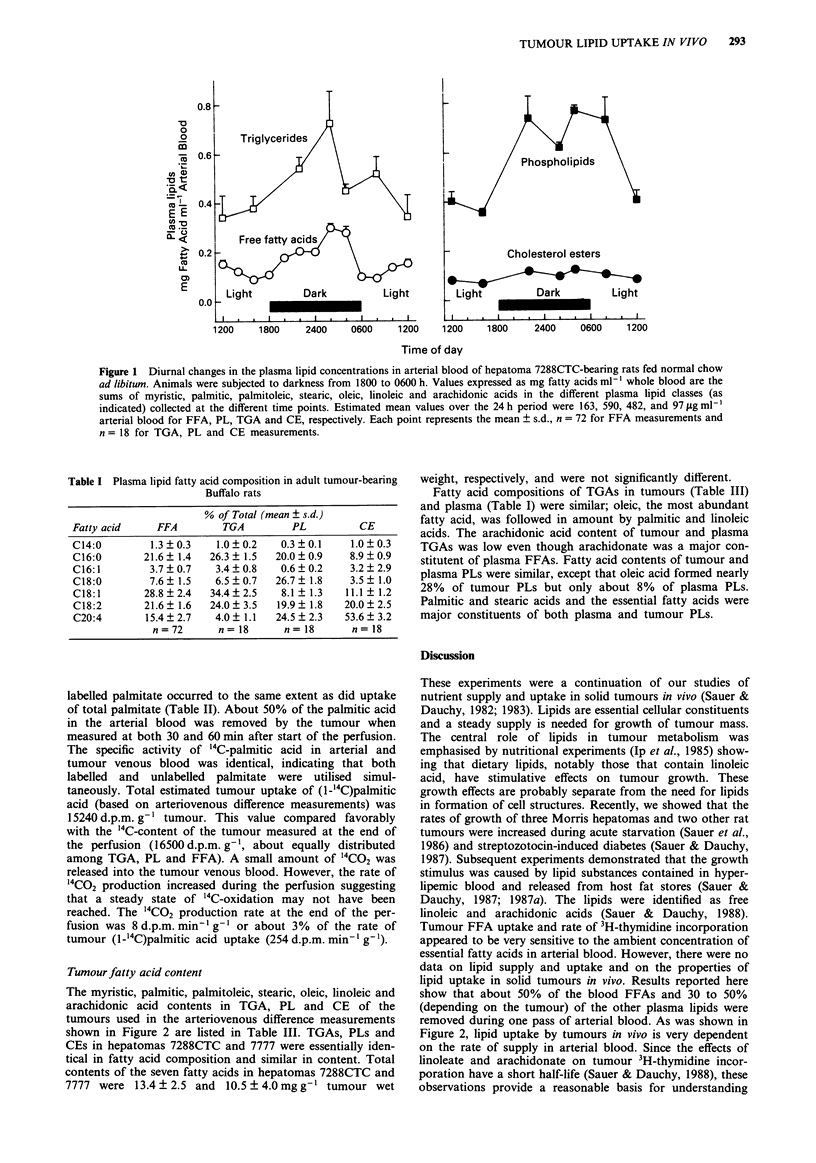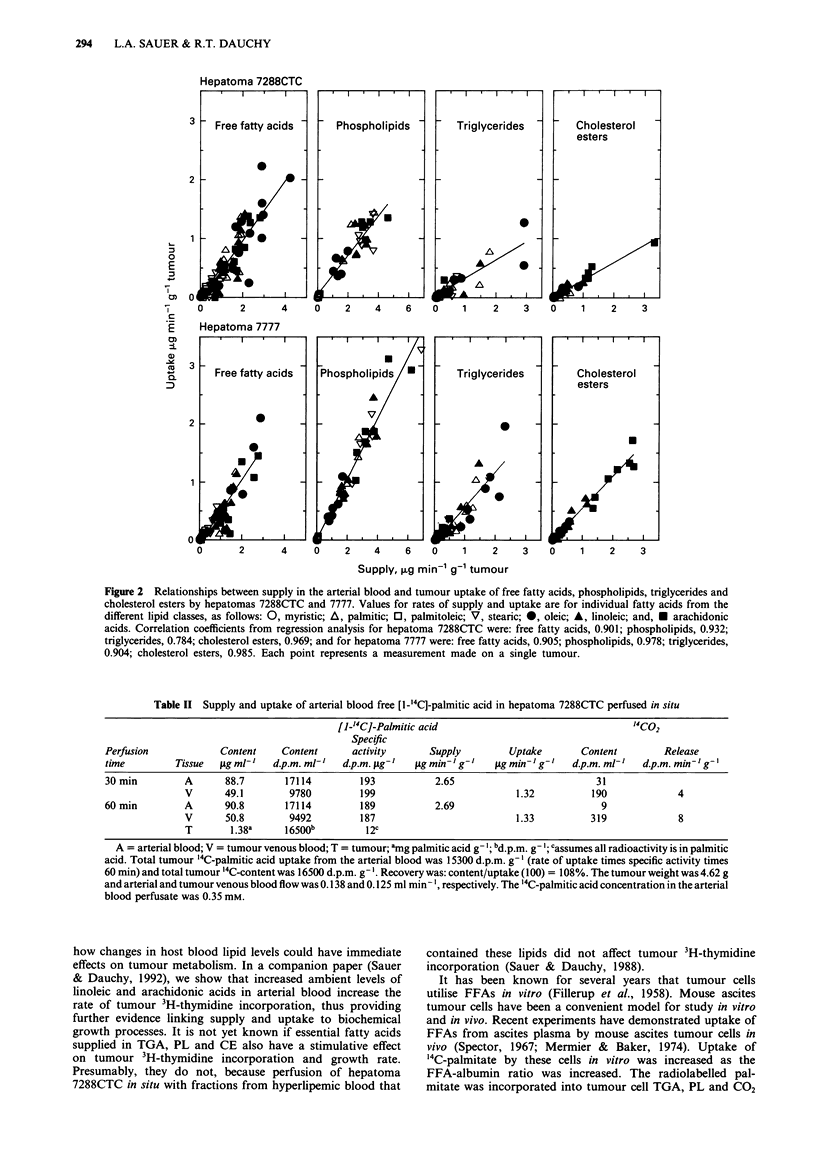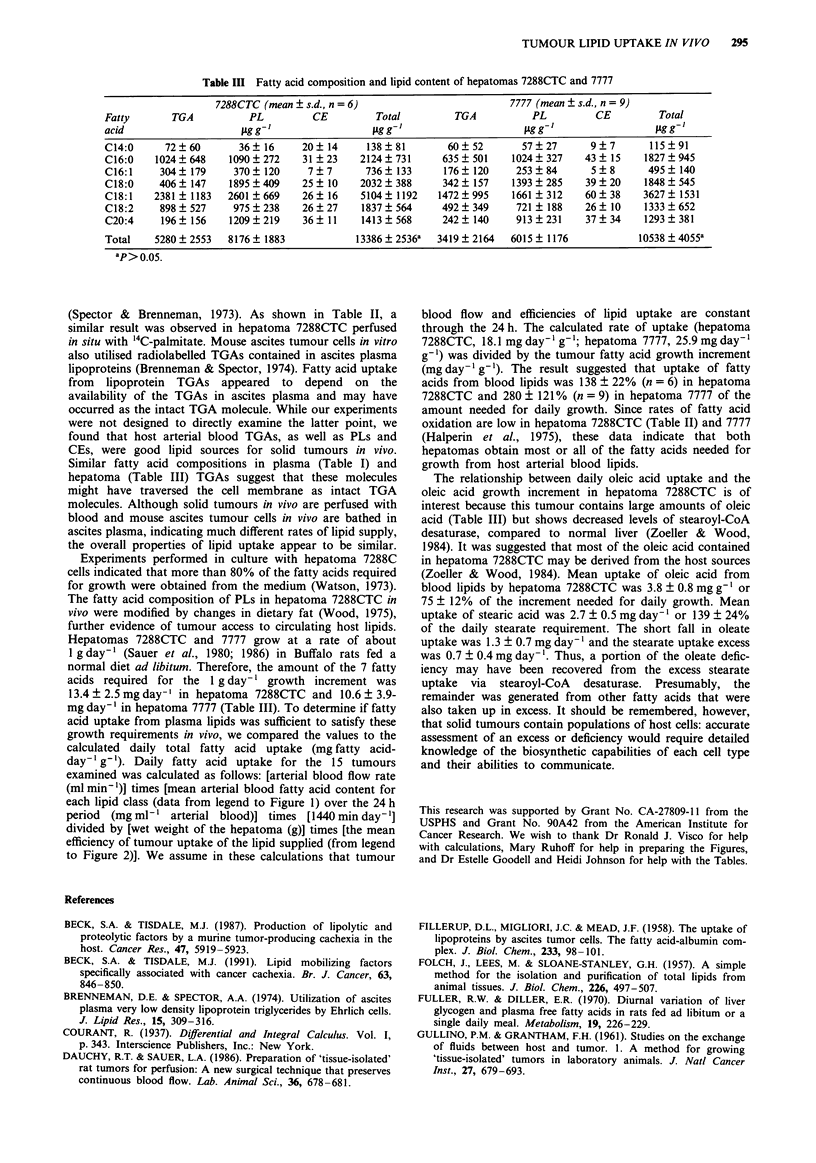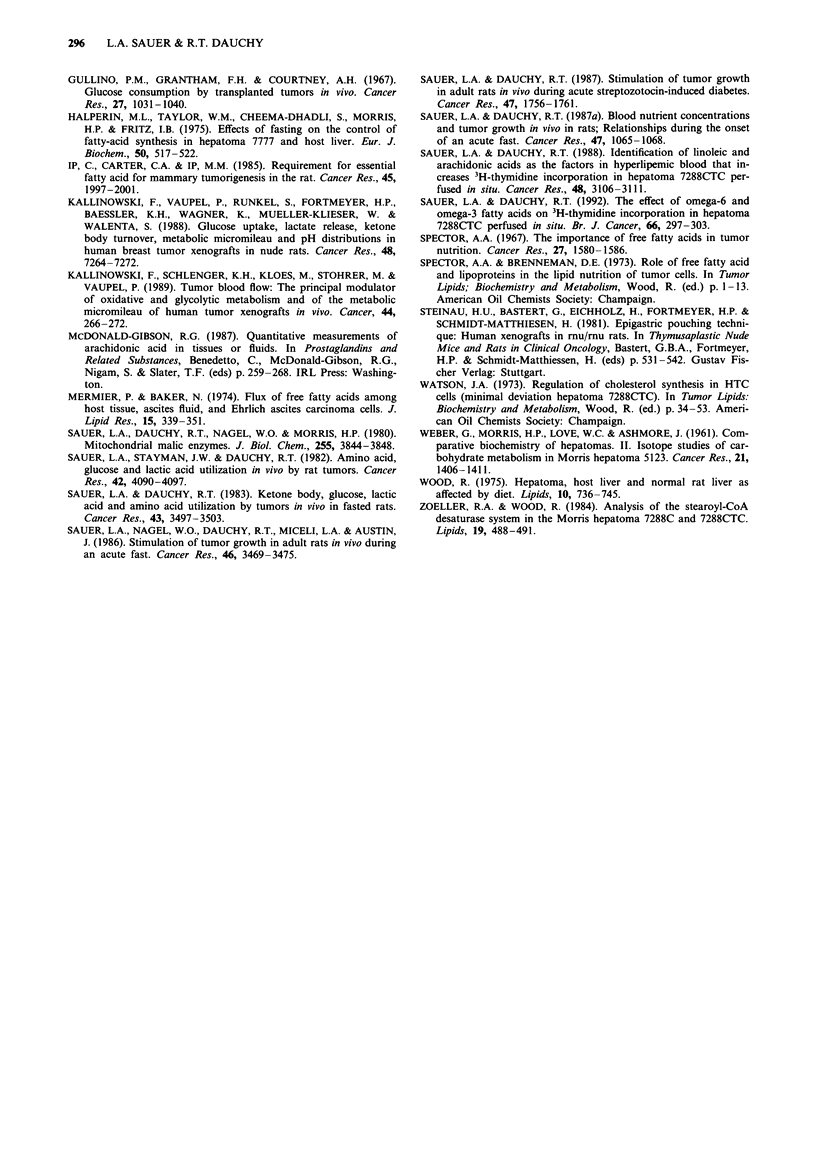# Uptake of plasma lipids by tissue-isolated hepatomas 7288CTC and 7777 in vivo.

**DOI:** 10.1038/bjc.1992.259

**Published:** 1992-08

**Authors:** L. A. Sauer, R. T. Dauchy

**Affiliations:** Cancer Research Laboratory, Mary Imogene Bassett Hospital, Cooperstown, New York 13326.

## Abstract

The uptake of myristic (C14:0), palmitic (C16:0), palmitoleic (C:16,N-7), stearic (C18:0), oleic (C18:1,N-9), linoleic (C18:2,N-6) and arachidonic (C20:4,N-6) acids from plasma free fatty acids (FFA), triglycerides (TGA), phospholipids (PL) and cholesterol esters (CE) was measured in tissue-isolated hepatomas 7288CTC and 7777 in vivo. Adult tumour-bearing Buffalo rats were fed a normal chow diet ad libitum and were subjected to darkness from 1800 to 0600 h. Arterial plasma levels of FFA, TGA, PL and CE were increased during the dark period without change in fatty acid compositions. Arteriovenous difference measurements of tumour lipid uptake were performed between 0600 and 0900 h and included both high (dark) and low (light) arterial blood lipid concentrations. The rate of lipid uptake from each lipid class was directly dependent on the rate of supply of the lipid to the tumour. The efficiency of uptake, however, depended on the type of plasma lipid and the tumour. During one pass of arterial blood, hepatoma 7288CTC (n = 5 to 13) removed 46, 33, 36 and 31%, and hepatoma 7777 (n = 7 to 9) removed 48, 50, 52 and 49% of the fatty acids supplied in FFA, TGA, PL and CE, respectively. Perfusion of tissue-isolated tumours in situ with donor blood containing plasma free (1-14C)palmitic acid showed that 14C-palmitic acid was removed from the arterial blood and was incorporated into tumour lipids and that 14CO2 was released into the tumour venous blood. Uptake of the seven fatty acids over a 24 h period was greatest from PL greater than TGA greater than FFA greater than CE and was estimated to total 18.1 +/- 3.5 mg fatty acids g-1 for hepatoma 7288CTC and 25.9 +/- 3.5 mg fatty acids g-1 for hepatoma 7777. Both hepatoma 7288CTC and 7777 grew at a rate of about 1 g day-1 and contained 13.4 +/- 2.5 and 10.6 +/- 3.9 mg of these 7 fatty acids g-1 tumour wet weight, respectively. We conclude that these two tumours obtain all of the fatty acids needed for daily growth from host arterial blood.


 © Macmillan Press Ltd., 1992

### Uptake of plasma lipids by tissue-isolated hepatomas 7288CTC and 7777 *in vivo*

L.A. Sauer & R.T. Dauchy

*Cancer Research Laboratory, Medical Research Institute, The Mary Imogene Bassett Hospital, Cooperstown, New York 13326, USA.*

**Summary** The uptake of myristic (C14:0), palmitic (C16:0), palmitoleic (C:16,N-7), stearic (C18:0), oleic (C18:1,N-9), linoleic (C18:2,N-6) and arachidonic (C20:4,N-6) acids from plasma free fatty acids (FFA), triglycerides (TGA), phospholipids (PL) and cholesterol esters (CE) was measured in tissue-isolated hepatomas 7288CTC and 7777 *in vivo*. Adult tumour-bearing Buffalo rats were fed a normal chow diet *ad libitum* and were subjected to darkness from 1800 to 0600 h. Arterial plasma levels of FFA, TGA, PL and CE were increased during the dark period without change in fatty acid compositions. Arteriovenous difference measurements of tumour lipid uptake were performed between 0600 and 0900 h and included both high (dark) and low (light) arterial blood lipid concentrations. The rate of lipid uptake from each lipid class was directly dependent on the rate of supply of the lipid to the tumour. The efficiency of uptake, however, depended on the type of plasma lipid and the tumour. During one pass of arterial blood, hepatoma 7288CTC ($n = 5$ to 13) removed 46, 33, 36 and 31%, and hepatoma 7777 ($n = 7$ to 9) removed 48, 50, 52 and 49% of the fatty acids supplied in FFA, TGA, PL and CE, respectively. Perfusion of tissue-isolated tumours *in situ* with donor blood containing plasma free (1-$^{14}$C)palmitic acid showed that $^{14}$C-palmitic acid was removed from the arterial blood and was incorporated into tumour lipids and that $^{14}CO_2$ was released into the tumour venous blood. Uptake of the seven fatty acids over a 24 h period was greatest from PL>TGA>FFA>CE and was estimated to total 18.1 ± 3.5 mg fatty acids g$^{-1}$ for hepatoma 7288CTC and 25.9 ± 3.5 mg fatty acids g$^{-1}$ for hepatoma 7777. Both hepatoma 7288CTC and 7777 grew at a rate of about 1 g day$^{-1}$ and contained 13.4 ± 2.5 and 10.6 ± 3.9 mg of these 7 fatty acids g$^{-1}$ tumour wet weight, respectively. We conclude that these two tumours obtain all of the fatty acids needed for daily growth from host arterial blood.

Experiments performed *in vivo* with tissue-isolated rat tumours (Gullino & Grantham, 1961; Sauer *et al.*, 1982) and with human tumour xenografts growing in nude rats (Steinau *et al.*, 1981) have demonstrated that solid tumours have a large capacity for uptake of host nutrients. Thirty to 40% of the glucose (Gullino *et al.*, 1967; Sauer *et al.*, 1982; Sauer & Dauchy, 1983; Kallinowski *et al.*, 1988), ketone bodies (Sauer & Dauchy, 1983; Kallinowski *et al.*, 1988) and amino acids (Sauer *et al.*, 1982) contained in arterial blood was removed during one pass through the tumour. The rates of nutrient uptake were directly dependent on the rates of supply and were not saturable at normal physiological blood concentrations (Sauer *et al.*, 1982; Kallinowski *et al.*, 1988). Arterial blood also transports lipid nutrients, including free fatty acids (FFA) and lipoproteins that contain triglycerides (TGA), phospholipids (PL) and cholesterol esters (CE). Solid tumours, especially those that are fast-growing and undifferentiated, are thought to have a diminished ability to synthesise fatty acids (Weber *et al.*, 1961) and, therefore, to obtain some or all of the lipids required for growth and metabolism from host sources. Tumours release lipolytic peptides that promote mobilisation of host lipid stores (Beck & Tisdale, 1987; 1991). Possibly, the function of the lipolytic agent is to increase the lipid supply to the tumour, including the essential fatty acids, which may have a stimulative effect on tumour growth (Sauer & Dauchy, 1988). Relationships between lipid supply in the arterial blood, tumour uptake and requirements for growth have not yet been investigated in solid tumours *in vivo*. In this study we measured uptake of seven fatty acids from plasma FFA, TGA, PL, and CE in tissue-isolated hepatomas 7288CTC and 7777. Total tumour fatty acid uptake was estimated for a 24 h period and was compared to the daily increment in tumour lipid content due to growth.

Correspondence: L.A. Sauer.
Received 12 February 1992; and in revised form 21 April 1992.

### Materials and methods

### *Reagents*

Hepatane (HPLC) grade, chloroform, methanol and ethanol were obtained from Fisher Chemical Co., Pittsburgh, PA. Heptane and chloroform were redistilled before use. Methyl esters of rapeseed oil fatty acids, standard FFAs and boron triflouride-methanol reagent were purchased from Supelco, Bellefonte, PA and from Sigma Chemical Co. St. Louis, MO. $^{14}$C-Palmitic acid (1-$^{14}$C, 56mCi mmol$^{-1}$) was purchased from NEN Research Products, Boston, MA. Butylated hydroxytoluene was obtained from Sigma Chemical Co.

### *Animals and diets*

The male and female Buffalo rats used in these experiments were obtained from colonies established here. Animals were maintained at 23°C in a 12 h light/dark cycle. Breeding pairs, pregnant females and experimental rats were fed a standard laboratory diet (Prolab mouse, rat and hamster 1000 formula; Agway, Inc., Syracuse, NY) and water *ad libitum*. Lipid analyses performed on different batches of this diet showed a mean fatty acid content of 39.2 mg g$^{-1}$, which was composed of 2.3% myristic (C14:0), 23.2% palmitic (C16:0), 2.8% palmitoleic (C16:1,N-7), 11.9% stearic (C18:0), 35.8% oleic (C18:1,N-9), 21.2% linoleic (C18:2,N-6) and 0.1% (arachidonic (C20:4,N-6) acids. Two additional unidentified fatty acids comprised about 3%. Ninety-one percent of the fatty acids was present as TGA and PL.

### *Tumour implantation and growth*

All experiments were performed with Morris hepatomas 7288CTC or 7777 grown subcutaneously as tissue-isolated tumours (Sauer *et al.*, 1982). Briefly, a 3-mm cube of tumour was attached to the end of a vascular stalk composed of the truncated left superficial inferior epigastric artery and vein; the femoral artery and vein distal to the sup. inf. epigastric vessels were not ligated (Dauchy & Sauer, 1986). The tumour implant and vascular stalk were enclosed in a Paraffin envelope (Gullino *et al*., 1961), placed in the inguinal fossa with a drop of sterile penicillin G procaine suspension (Wyeth Laboratories, Inc., Philadelphia, PA), and the skin incision was closed. Arterial blood supply to and venous drainage from the implant were established through the epigastric vessels. Vascular connections to other host tissues were blocked by the Paraffin envelope. Tumour weights *in vivo* were estimated from measurements made through the skin (Sauer *et al*., 1986).

Two male and three female tumour-bearing rats weighing 250 to 300 g were anticoagulated with warfarin for 4 to 9 days before tumour harvest. Pellets of normal diet and Coumadin tablets (Du Pont Pharmaceuticals, Wilmington, DE) were ground together to give a mixture that contained 1.25 μg crystalline sodium warfarin $g^{-1}$ diet. Each rat ate 18 to 22 g of this diet for a dose of about 0.1 mg $kg^{-1}$ body weight daily.

*Collection of arterial blood*

Adult Buffalo rats bearing tissue-isolated hepatoma 7288CTC or 7777 were anaesthetised by $CO_2$ inhalation and blood was collected by heart puncture. Samples were obtained from groups of three to six rats at 2 h (FFA analysis) or 4 to 6 h (TGA, PL and CE analyses) intervals for a total of 24 h. Blood collection times were adjusted so that no animal was sampled more frequently than once per day.

*Arteriovenous difference measurements*

Tissue-isolated hepatomas weighing 3.2 to 8 g (mean = 5.41 ± 0.96 g, $n = 22$) were prepared for arteriovenous difference measurements (Sauer *et al*., 1982) using the modifications described by Dauchy & Sauer (1986). Host carcass weights were 306 ± 42 g ($n = 11$) for male rats and 252 ± 14 g ($n = 11$) for female rats. All animals were anaesthesised with pentobarbital (25 mg $kg^{-1}$ body weight, IP) and breathed air during the procedure. After exposure of the tumour and tumour vasculature, the femoral artery and vein distal to the tumour were ligated. The tumour was inspected to determine that there were no vascular connections other than the sup. inf. epigastric vessels and the host was anticoagulated by injecting 200 units of sodium heparin into the jugular vein. (Heparin injection was not necessary in measurements made in warfarin-treated host rats.) A catheter was placed in the left carotid artery and a butterfly catheter (no. 4573, Abbott Hospitals, North Chicago, IL) was inserted into the vein draining the tumour. Blood flowed passively from the venous catheter; flow rates were calculated from timed collections (Sauer *et al*., 1982). Arterial and tumour venous blood samples (about 0.5 ml) were collected simultaneously. The samples were immediately chilled in ice, then centrifuged and the plasma removed and stored in ice in capped tubes. Hematocrits were measured on blood obtained directly from the arterial and tumour venous catheters. Plasma protein contents were assayed using a biuret method.

Tumours were removed from the host rats, chilled in ice-cold 0.15 M NaCl, cut in two and weighed. Three tumours were hemorrhagic and were discarded. Tissue-isolated tumours grown on the sup. inf. epigastric vessels generally show only microscopic necrosis (Sauer *et al*., 1982). However, a portion of the original tumour implant (3 mm cube) often becomes necrotic. Since these necrotic volumes were small and could not be easily removed without disaggregating the tumour, they were included in the total tumour weight. A portion of the tumour was minced, visibly necrotic fragments were removed and a 20% homogenate (w/v) was prepared in 0.15 M NaCl containing 0.05% butylated hydroxytoluene. All procedures were performed at 0 to 4°C.

Tumour substrate supply rates were calculated by multiplying the arterial whole blood concentration by the arterial blood flow rate and dividing by the tumour wet weight. Outflow rates were calculated by multiplying the substrate concentration in tumour venous whole blood by the tumour venous blood flow rate and dividing by the tumour wet weight. Since the total tumour wet weight included small necrotic volumes, which presumably had no blood flow, these supply and outflow rates may be slightly lower than the actual rates. Uptake was the difference between supply and outflow. Both supply and uptake rates have units of μg (or mg) fatty acid $min^{-1}$ $g^{-1}$ wet weight tumour. These units were selected because the mass of fatty acid uptake is related directly to the tumour mass. Molar quantities may be obtained after division by the molecular weight of the fatty acid.

*Tumour perfusion* in situ

Tissue-isolated hepatomas used for perfusion were implanted and prepared for perfusion as described above. The femoral artery and vein distal to the tumour were not ligated until after insertion of the butterfly catheter into the tumour vein and insertion of the arterial catheter (carrying the donor blood perfusate) into the femoral-epigastric arterial trunk leading to the tumour. When flow of donor blood was established, the host was exsanguinated through the carotid catheter. Temperature of the host body was monitored using a rectal probe and maintained at 37°C with a heating pad beneath and a heat lamp above the animal.

Arterial blood used for perfusion was collected from the carotid arteries of adult, heparinised male or female rats fed normal diet *ad libitum*. Donor rats were anaesthetised with pentobarbital (25 mg $kg^{-1}$) before exsanguination. Pooled donor blood was filtered through two layers of cheesecloth and separated into plasma and cells by centrifugation. Carrier-free $^{14}C$-palmitic acid (sufficient to give about 20,000 dpm $ml^{-1}$ reconstituted whole blood) was added to a plastic tube and the solvent (ethanol) air dried. The donor plasma was added and mixed by gentle stirring at 0°C for about 20 min. Small portions of the plasma were counted to determine entry of the labelled palmitic acid into the plasma. Cells from the donor blood were added back, mixed, and the suspension transferred to a plastic container immersed in ice and stirred with a magnetic stirrer. The labelled whole blood perfusate was pumped through the tumour with a peristaltic pump (Harvard Apparatus, Natick, MA) at a setting adjusted to give a flow rate from the tumour venous catheter of about 0.12 ml $min^{-1}$. The perfusate was warmed in a 37°C water bath immediately before entering the tumour. pH, $pCO_2$ and $pO_2$ in the arterial blood perfusate were maintained at about 7.4 and 40 and 100 mm Hg, respectively, by gently blowing a water-saturated air-$CO_2$ mixture over the surface of the stirred perfusate. Measurements were made using a blood gas analyser (Model 945, AVL, Graz, Austria). Arterial blood samples were collected from a Y-tube connector located in the arterial catheter immediately before the tumour.

The specific activity of $^{14}C$-palmitic acid in arterial and tumour venous blood was determined by analysing and counting plasma FFA extracts. No radioactivity was found in any other plasma lipid fraction. $^{14}CO_2$ contained in arterial and tumour venous blood was measured in closed flasks using phenethylamine as trapping agent (Sauer *et al*., 1980). Incorporation of $^{14}C$-palmitic acid into tumour lipids was measured in a total lipid extract and in a portion of tumour solubilised in Protosol (NEN Research Products, Boston, MA). Radioactivity was counted in a scintillation counter. Addition of $^{14}C$-palmitic acid directly to the donor plasma avoided addition of exogenous albumin (or other proteins) that might either interfere with or modify tumour fatty acid uptake. The exogenous $^{14}C$-palmitic acid in donor plasma migrated with the albumin band during electrophoresis.

*Lipid extraction and chromatography*

FFAs were extracted from the arterial and tumour venous plasma samples as previously described (Sauer & Dauchy, 1988). Heptadecanoic acid was used as an internal standard and was added to the plasma prior to extraction. Methyl esters of the FFAs were formed using boron trifluoride-methanol reagent. Arterial and tumour venous plasma TGA, PL and CE were extracted from 0.2 ml of plasma by the Folch method (Folch *et al.*, 1957), using the procedure of McDonald-Gibson (McDonald-Gibson, 1987). Internal standard, dissolved in chloroform, was added following the initial mixing of the plasma sample with methanol and prior to the addition of chloroform. In some experiments the lipid extract was separated by thin layer chromatography on silica gel G plates (Redi-plates, Fisher Scientific, Pittsburgh, PA) that were sprayed with Rhodamine B in ethanol and activated at 100°C. The solvent mixture was n-hexane:diethyl ether: glacial acetic acid (80:20:2 by volume). Heptadecanoic acid, tripentadecanoin, diheptadecanoyl phosphatidyl choline, and cholesterol heptadecanoate were used as internal standards. The total plasma lipid extract or the separated lipid classes were saponified in methanolic-NaOH (0.5 M) for 5 min at 100°C and the fatty acids methylated using boron triflouride-methanol reagent for 2 min at 100°C. Fatty acid contents are given as μg (or mg) $ml^{-1}$ whole blood or as percent of total fatty acids. Molar concentrations are obtained after division by the fatty acid molecular weight.

Tumour lipids were extracted from 0.25 ml of homogenate and were analysed directly or were separated by thin layer chromatography as described above. Homogenates were kept at 0°C to slow hydrolysis of TGAs by tumour lipases. Fatty acid contents are given as μg (or mg) $g^{-1}$ tumour wet weight. All gas chromatographic analyses of blood and tumour samples were performed in duplicate.

*Fatty acid analysis*

The fatty acid methyl esters were measured using a Hewlett-Packard Model 5890A gas chromatograph equipped with a flame-ionisation detector, an electronic integrator (Model 3396A) and autoinjector (Model 7673A). Separations were performed with a 0.25 mm × 30 m capillary column (Model 2330, Supelco Inc., Bellefonte, PA) at 190°C with helium as the carrier gas (linear gas rate: 19 cm $sec^{-1}$; split, 100:1). Injection port and detector were at 220°C. Fatty acid methyl esters were identified by their retention times compared to known standards.

*Statistical analysis*

Means were presented ± 1s.d. and were compared by Student's *t*-test or by one-way analysis of variance and the Duncan multiple range test. $P < 0.05$ was considered significant.

### Results

*Characteristics of arterial and tumour venous blood*

The plasma protein concentrations (53.3 ± 6.9 g $l^{-1}$, $n = 13$) and hematocrits (41.3 ± 4.9%, $n = 21$) of arterial blood were increased during passage through hepatomas 7288CTC and 7777 *in vivo* and during perfusion *in situ*. Mean values for plasma protein concentration and hematocrit in tumour venous blood were 61.6 ± 7.3 g $l^{-1}$ ($P < 0.01$) and 46.9 ± 4.7% ($P < 0.01$), respectively, indicating that about 15% of arterial blood water was lost in one pass through the tumour. The excess fluid was apparently drained away by lymphatic vessels in the subcutaneous space. Arterial blood flow rates were corrected for this fluid loss by multiplying the measured tumour venous blood flow rates by the quotient of either the tumour venous blood hematocrit/arterial blood hematocrit or the tumour venous plasma protein concentration/arterial blood plasma protein concentration. Calculated arterial blood flow rates were 0.141 ± 0.01 ml $min^{-1}$; mean tumour venous blood flow rates were 0.124 ± 0.01 ($n = 22$) and ranged from 0.11 to 0.133 ml $min^{-1}$ indicating the passive flow from the venous catheter was reproducible from tumour to tumour. Total blood flow in these tissue-isolated hepatomas appears to depend on the normal, nearly constant flow in the sup. inf. epigastric artery. Consequently, larger tumours have a lower blood flow rate per unit mass (ml $min^{-1}$ $g^{-1}$ tumour) than smaller tumours. Similar passive tumour venous blood flow rates were observed by Kallinowski *et al.* (1989) during arterioenous difference measurements across tissue-isolated human tumour xenografts growing on the sup. inf. epigastric artery of nude rats. These authors also observed, however, that the rate of passive venous flow was a function of the tumour type. pH, $pO_2$ and $pCO_2$ ($n = 12$) were 7.41 ± 0.06, and 88.8 ± 11.4 and 27.4 ± 7.2 mm Hg in host arterial blood, respectively, and 7.35 ± 0.05, and 23.8 ± 8.9 and 49.6 ± 6.2 mm Hg in tumour venous blood, respectively. Similar differences in hematocrit, pH and blood gases were observed across tissue-isolated human tumour xenografts *in vivo* in nude rats (Kallinowski *et al.*, 1989).

*Arterial plasma lipids*

Diurnal rhythms in amounts of arterial blood plasma lipids were observed in tumour-bearing Buffalo rats (Figure 1). The sums of myristic, palmitic, palmitoleic, stearic, oleic, linoleic and arachidonic acids contained in FFA, TGA, PL and CE were low during the light period and increased between 1600 to 2000 h with onset of darkness. Plasma lipid fatty acid contents measured at 1600 h were significantly less ($P < 0.05$) than those measured at 2200 h. The content of plasma FFAs showed a second, small increase during mid-day. Diurnal variations in blood lipid concentrations resulted from increased feeding during the dark period (Fuller & Diller, 1970). Areas under the curves shown in Figure 1 were calculated using the trapezoidal rule (Courant, 1937), which estimated mean values over the 24 h period of 163, 590, 482 and 97 μg fatty acid $ml^{-1}$ whole arterial blood for FFA, PL, TGA and CE, respectively.

Despite the large diurnal variation in plasma lipid content, the fatty acid compositions of individual arterial blood plasma lipids were remarkably constant (Table I). Plasma FFA and TGA were good sources of oleic, palmitic and linoleic acids. FFA (but not TGA) also contained substantial amounts of arachidonic acid. Stearic, arachidonic, palmitic and linoleic acids comprised over 90% of the fatty acid content of plasma PL, the most abundant arterial plasma lipid. Stearic acid was higher in PL than in any other plasma lipid. Although CE was the least abundant plasma lipid, it was a good source of arachidonic and linoleic acids.

*Uptake of arterial plasma lipids*

The relationships between supply of myristic, palmitic, palmitoleic, stearic, oleic, linoleic and arachidonic acids in FFA, TGA, PL and CE and uptake by hepatomas 7288CTC and 7777 *in vivo* are shown in Figure 2. Fatty acid uptake was directly dependent on the rate of supply of the lipid. As judged from the regression lines, hepatoma 7288CTC removed 46.2, 36.0, 32.6 and 31.4% and hepatoma 7777 removed 47.7, 52.4, 50.0 and 48.6% of the seven fatty acids contained in FFA, PL, TGA and CE, respectively, during one pass of arterial blood. Uptake of individual fatty acids depended on the lipid supply to the tumour and on the fatty acid composition of the lipid. For example, stearic acid is a major constituent of PL and uptake of stearate from PL occurred at a substantial rate, especially during the dark period. It cannot be decided from these data if the intact TGA, PL or CE molecule was taken up by the tumour or if the fatty acids were first released and then accumulated. Arteriovenous difference measurements performed in coumadin-treated rats were not different from measurements made in rats anticoagulated with heparin and the data were combined; heparin-treatment did not modify tumour lipid uptake.

*Perfusion* in situ

Perfusion of hepatoma 7288CTC *in situ* with donor blood containing $^{14}C$-palmitic acid showed that the uptake of

**Figure 1** Diurnal changes in the plasma lipid concentrations in arterial blood of hepatoma 7288CTC-bearing rats fed normal chow *ad libitum*. Animals were subjected to darkness from 1800 to 0600 h. Values expressed as mg fatty acids $ml^{-1}$ whole blood are the sums of myristic, palmitic, palmitoleic, stearic, oleic, linoleic and arachidonic acids in the different plasma lipid classes (as indicated) collected at the different time points. Estimated mean values over the 24 h period were 163, 590, 482, and 97 $\mu g\ ml^{-1}$ arterial blood for FFA, PL, TGA and CE, respectively. Each point represents the mean ± s.d., $n = 72$ for FFA measurements and $n = 18$ for TGA, PL and CE measurements.

**Table I** Plasma lipid fatty acid composition in adult tumour-bearing Buffalo rats

| | *% of Total (mean ± s.d.)* | | | |
|---|---|---|---|---|
| *Fatty acid* | *FFA* | *TGA* | *PL* | *CE* |
| C14:0 | 1.3 ± 0.3 | 1.0 ± 0.2 | 0.3 ± 0.1 | 1.0 ± 0.3 |
| C16:0 | 21.6 ± 1.4 | 26.3 ± 1.5 | 20.0 ± 0.9 | 8.9 ± 0.9 |
| C16:1 | 3.7 ± 0.7 | 3.4 ± 0.8 | 0.6 ± 0.2 | 3.2 ± 2.9 |
| C18:0 | 7.6 ± 1.5 | 6.5 ± 0.7 | 26.7 ± 1.8 | 3.5 ± 1.0 |
| C18:1 | 28.8 ± 2.4 | 34.4 ± 2.5 | 8.1 ± 1.3 | 11.1 ± 1.2 |
| C18:2 | 21.6 ± 1.6 | 24.0 ± 3.5 | 19.9 ± 1.8 | 20.0 ± 2.5 |
| C20:4 | 15.4 ± 2.7 | 4.0 ± 1.1 | 24.5 ± 2.3 | 53.6 ± 3.2 |
| | $n = 72$ | $n = 18$ | $n = 18$ | $n = 18$ |

labelled palmitate occurred to the same extent as did uptake of total palmitate (Table II). About 50% of the palmitic acid in the arterial blood was removed by the tumour when measured at both 30 and 60 min after start of the perfusion. The specific activity of $^{14}C$-palmitic acid in arterial and tumour venous blood was identical, indicating that both labelled and unlabelled palmitate were utilised simultaneously. Total estimated tumour uptake of (1-$^{14}C$)palmitic acid (based on arteriovenous difference measurements) was 15240 d.p.m. $g^{-1}$ tumour. This value compared favorably with the $^{14}C$-content of the tumour measured at the end of the perfusion (16500 d.p.m. $g^{-1}$, about equally distributed among TGA, PL and FFA). A small amount of $^{14}CO_2$ was released into the tumour venous blood. However, the rate of $^{14}CO_2$ production increased during the perfusion suggesting that a steady state of $^{14}C$-oxidation may not have been reached. The $^{14}CO_2$ production rate at the end of the perfusion was 8 d.p.m. $min^{-1}$ $g^{-1}$ or about 3% of the rate of tumour (1-$^{14}C$)palmitic acid uptake (254 d.p.m. $min^{-1}$ $g^{-1}$).

### *Tumour fatty acid content*

The myristic, palmitic, palmitoleic, stearic, oleic, linoleic and arachidonic acid contents in TGA, PL and CE of the tumours used in the arteriovenous difference measurements shown in Figure 2 are listed in Table III. TGAs, PLs and CEs in hepatomas 7288CTC and 7777 were essentially identical in fatty acid composition and similar in content. Total contents of the seven fatty acids in hepatomas 7288CTC and 7777 were 13.4 ± 2.5 and 10.5 ± 4.0 mg $g^{-1}$ tumour wet weight, respectively, and were not significantly different.

Fatty acid compositions of TGAs in tumours (Table III) and plasma (Table I) were similar; oleic, the most abundant fatty acid, was followed in amount by palmitic and linoleic acids. The arachidonic acid content of tumour and plasma TGAs was low even though arachidonate was a major constitutent of plasma FFAs. Fatty acid contents of tumour and plasma PLs were similar, except that oleic acid formed nearly 28% of tumour PLs but only about 8% of plasma PLs. Palmitic and stearic acids and the essential fatty acids were major constituents of both plasma and tumour PLs.

### Discussion

These experiments were a continuation of our studies of nutrient supply and uptake in solid tumours *in vivo* (Sauer & Dauchy, 1982; 1983). Lipids are essential cellular constituents and a steady supply is needed for growth of tumour mass. The central role of lipids in tumour metabolism was emphasised by nutritional experiments (Ip *et al.*, 1985) showing that dietary lipids, notably those that contain linoleic acid, have stimulative effects on tumour growth. These growth effects are probably separate from the need for lipids in formation of cell structures. Recently, we showed that the rates of growth of three Morris hepatomas and two other rat tumours were increased during acute starvation (Sauer *et al.*, 1986) and streptozotocin-induced diabetes (Sauer & Dauchy, 1987). Subsequent experiments demonstrated that the growth stimulus was caused by lipid substances contained in hyperlipemic blood and released from host fat stores (Sauer & Dauchy, 1987; 1987*a*). The lipids were identified as free linoleic and arachidonic acids (Sauer & Dauchy, 1988). Tumour FFA uptake and rate of $^{3}H$-thymidine incorporation appeared to be very sensitive to the ambient concentration of essential fatty acids in arterial blood. However, there were no data on lipid supply and uptake and on the properties of lipid uptake in solid tumours *in vivo*. Results reported here show that about 50% of the blood FFAs and 30 to 50% (depending on the tumour) of the other plasma lipids were removed during one pass of arterial blood. As was shown in Figure 2, lipid uptake by tumours *in vivo* is very dependent on the rate of supply in arterial blood. Since the effects of linoleate and arachidonate on tumour $^{3}H$-thymidine incorporation have a short half-life (Sauer & Dauchy, 1988), these observations provide a reasonable basis for understanding

**Figure 2** Relationships between supply in the arterial blood and tumour uptake of free fatty acids, phospholipids, triglycerides and cholesterol esters by hepatomas 7288CTC and 7777. Values for rates of supply and uptake are for individual fatty acids from the different lipid classes, as follows: O, myristic; Δ, palmitic; □, palmitoleic; ∇, stearic; ●, oleic; ▲, linoleic; and, ■ arachidonic acids. Correlation coefficients from regression analysis for hepatoma 7288CTC were: free fatty acids, 0.901; phospholipids, 0.932; triglycerides, 0.784; cholesterol esters, 0.969; and for hepatoma 7777 were: free fatty acids, 0.905; phospholipids, 0.978; triglycerides, 0.904; cholesterol esters, 0.985. Each point represents a measurement made on a single tumour.

**Table II** Supply and uptake of arterial blood free [1-$^{14}$C]-palmitic acid in hepatoma 7288CTC perfused *in situ*

| | | *[1-$^{14}$C]-Palmitic acid* | | | | | *$^{14}CO_2$* | |
|---|---|---|---|---|---|---|---|---|
| *Perfusion time* | *Tissue* | *Content µg ml$^{-1}$* | *Content d.p.m. ml$^{-1}$* | *Specific activity d.p.m. µg$^{-1}$* | *Supply µg min$^{-1}$ g$^{-1}$* | *Uptake µg min$^{-1}$ g$^{-1}$* | *Content d.p.m. ml$^{-1}$* | *Release d.p.m. min$^{-1}$ g$^{-1}$* |
| 30 min | A | 88.7 | 17114 | 193 | 2.65 | | 31 | |
| | V | 49.1 | 9780 | 199 | | 1.32 | 190 | 4 |
| 60 min | A | 90.8 | 17114 | 189 | 2.69 | | 9 | |
| | V | 50.8 | 9492 | 187 | | 1.33 | 319 | 8 |
| | T | 1.38[a] | 16500[b] | 12[c] | | | | |

A = arterial blood; V = tumour venous blood; T = tumour; [a]mg palmitic acid g$^{-1}$; [b]d.p.m. g$^{-1}$; [c]assumes all radioactivity is in palmitic acid. Total tumour $^{14}$C-palmitic acid uptake from the arterial blood was 15300 d.p.m. g$^{-1}$ (rate of uptake times specific activity times 60 min) and total tumour $^{14}$C-content was 16500 d.p.m. g$^{-1}$. Recovery was: content/uptake (100) = 108%. The tumour weight was 4.62 g and arterial and tumour venous blood flow was 0.138 and 0.125 ml min$^{-1}$, respectively. The $^{14}$C-palmitic acid concentration in the arterial blood perfusate was 0.35 mM.

how changes in host blood lipid levels could have immediate effects on tumour metabolism. In a companion paper (Sauer & Dauchy, 1992), we show that increased ambient levels of linoleic and arachidonic acids in arterial blood increase the rate of tumour $^{3}$H-thymidine incorporation, thus providing further evidence linking supply and uptake to biochemical growth processes. It is not yet known if essential fatty acids supplied in TGA, PL and CE also have a stimulative effect on tumour $^{3}$H-thymidine incorporation and growth rate. Presumably, they do not, because perfusion of hepatoma 7288CTC *in situ* with fractions from hyperlipemic blood that contained these lipids did not affect tumour $^{3}$H-thymidine incorporation (Sauer & Dauchy, 1988).

It has been known for several years that tumour cells utilise FFAs *in vitro* (Fillerup *et al.*, 1958). Mouse ascites tumour cells have been a convenient model for study *in vitro* and *in vivo*. Recent experiments have demonstrated uptake of FFAs from ascites plasma by mouse ascites tumour cells *in vivo* (Spector, 1967; Mermier & Baker, 1974). Uptake of $^{14}$C-palmitate by these cells *in vitro* was increased as the FFA-albumin ratio was increased. The radiolabelled palmitate was incorporated into tumour cell TGA, PL and $CO_2$

**Table III** Fatty acid composition and lipid content of hepatomas 7288CTC and 7777

| *Fatty acid* | *7288CTC (mean ± s.d., n = 6)* TGA | PL $\mu g\ g^{-1}$ | CE | *Total* $\mu g\ g^{-1}$ | *7777 (mean ± s.d., n = 9)* TGA | PL $\mu g\ g^{-1}$ | CE | *Total* $\mu g\ g^{-1}$ |
|---|---|---|---|---|---|---|---|---|
| C14:0 | 72 ± 60 | 36 ± 16 | 20 ± 14 | 138 ± 81 | 60 ± 52 | 57 ± 27 | 9 ± 7 | 115 ± 91 |
| C16:0 | 1024 ± 648 | 1090 ± 272 | 31 ± 23 | 2124 ± 731 | 635 ± 501 | 1024 ± 327 | 43 ± 15 | 1827 ± 945 |
| C16:1 | 304 ± 179 | 370 ± 120 | 7 ± 7 | 736 ± 133 | 176 ± 120 | 253 ± 84 | 5 ± 8 | 495 ± 140 |
| C18:0 | 406 ± 147 | 1895 ± 409 | 25 ± 10 | 2032 ± 388 | 342 ± 157 | 1393 ± 285 | 39 ± 20 | 1848 ± 545 |
| C18:1 | 2381 ± 1183 | 2601 ± 669 | 26 ± 16 | 5104 ± 1192 | 1472 ± 995 | 1661 ± 312 | 60 ± 38 | 3627 ± 1531 |
| C18:2 | 898 ± 527 | 975 ± 238 | 26 ± 27 | 1837 ± 564 | 492 ± 349 | 721 ± 188 | 26 ± 10 | 1333 ± 652 |
| C20:4 | 196 ± 156 | 1209 ± 219 | 36 ± 11 | 1413 ± 568 | 242 ± 140 | 913 ± 231 | 37 ± 34 | 1293 ± 381 |
| Total | 5280 ± 2553 | 8176 ± 1883 | | 13386 ± 2536[a] | 3419 ± 2164 | 6015 ± 1176 | | 10538 ± 4055[a] |

[a] $P > 0.05$.

(Spector & Brenneman, 1973). As shown in Table II, a similar result was observed in hepatoma 7288CTC perfused *in situ* with $^{14}$C-palmitate. Mouse ascites tumour cells *in vitro* also utilised radiolabelled TGAs contained in ascites plasma lipoproteins (Brenneman & Spector, 1974). Fatty acid uptake from lipoprotein TGAs appeared to depend on the availability of the TGAs in ascites plasma and may have occurred as the intact TGA molecule. While our experiments were not designed to directly examine the latter point, we found that host arterial blood TGAs, as well as PLs and CEs, were good lipid sources for solid tumours *in vivo*. Similar fatty acid compositions in plasma (Table I) and hepatoma (Table III) TGAs suggest that these molecules might have traversed the cell membrane as intact TGA molecules. Although solid tumours *in vivo* are perfused with blood and mouse ascites tumour cells *in vivo* are bathed in ascites plasma, indicating much different rates of lipid supply, the overall properties of lipid uptake appear to be similar.

Experiments performed in culture with hepatoma 7288C cells indicated that more than 80% of the fatty acids required for growth were obtained from the medium (Watson, 1973). The fatty acid composition of PLs in hepatoma 7288CTC *in vivo* were modified by changes in dietary fat (Wood, 1975), further evidence of tumour access to circulating host lipids. Hepatomas 7288CTC and 7777 grow at a rate of about 1 g day$^{-1}$ (Sauer *et al.*, 1980; 1986) in Buffalo rats fed a normal diet *ad libitum*. Therefore, the amount of the 7 fatty acids required for the 1 g day$^{-1}$ growth increment was 13.4 ± 2.5 mg day$^{-1}$ in hepatoma 7288CTC and 10.6 ± 3.9-mg day$^{-1}$ in hepatoma 7777 (Table III). To determine if fatty acid uptake from plasma lipids was sufficient to satisfy these growth requirements *in vivo*, we compared the values to the calculated daily total fatty acid uptake (mg fatty acid-day$^{-1}$ g$^{-1}$). Daily fatty acid uptake for the 15 tumours examined was calculated as follows: [arterial blood flow rate (ml min$^{-1}$)] times [mean arterial blood fatty acid content for each lipid class (data from legend to Figure 1) over the 24 h period (mg ml$^{-1}$ arterial blood)] times [1440 min day$^{-1}$] divided by [wet weight of the hepatoma (g)] times [the mean efficiency of tumour uptake of the lipid supplied (from legend to Figure 2)]. We assume in these calculations that tumour blood flow and efficiencies of lipid uptake are constant through the 24 h. The calculated rate of uptake (hepatoma 7288CTC, 18.1 mg day$^{-1}$ g$^{-1}$; hepatoma 7777, 25.9 mg day$^{-1}$ g$^{-1}$) was divided by the tumour fatty acid growth increment (mg day$^{-1}$ g$^{-1}$). The result suggested that uptake of fatty acids from blood lipids was 138 ± 22% ($n = 6$) in hepatoma 7288CTC and 280 ± 121% ($n = 9$) in hepatoma 7777 of the amount needed for daily growth. Since rates of fatty acid oxidation are low in hepatoma 7288CTC (Table II) and 7777 (Halperin *et al.*, 1975), these data indicate that both hepatomas obtain most or all of the fatty acids needed for growth from host arterial blood lipids.

The relationship between daily oleic acid uptake and the oleic acid growth increment in hepatoma 7288CTC is of interest because this tumour contains large amounts of oleic acid (Table III) but shows decreased levels of stearoyl-CoA desaturase, compared to normal liver (Zoeller & Wood, 1984). It was suggested that most of the oleic acid contained in hepatoma 7288CTC may be derived from the host sources (Zoeller & Wood, 1984). Mean uptake of oleic acid from blood lipids by hepatoma 7288CTC was 3.8 ± 0.8 mg g$^{-1}$ or 75 ± 12% of the increment needed for daily growth. Mean uptake of stearic acid was 2.7 ± 0.5 mg day$^{-1}$ or 139 ± 24% of the daily stearate requirement. The short fall in oleate uptake was 1.3 ± 0.7 mg day$^{-1}$ and the stearate uptake excess was 0.7 ± 0.4 mg day$^{-1}$. Thus, a portion of the oleate deficiency may have been recovered from the excess stearate uptake via stearoyl-CoA desaturase. Presumably, the remainder was generated from other fatty acids that were also taken up in excess. It should be remembered, however, that solid tumours contain populations of host cells: accurate assessment of an excess or deficiency would require detailed knowledge of the biosynthetic capabilities of each cell type and their abilities to communicate.

This research was supported by Grant No. CA-27809-11 from the USPHS and Grant No. 90A42 from the American Institute for Cancer Research. We wish to thank Dr Ronald J. Visco for help with calculations, Mary Ruhoff for help in preparing the Figures, and Dr Estelle Goodell and Heidi Johnson for help with the Tables.

### References

BECK, S.A. & TISDALE, M.J. (1987). Production of lipolytic and proteolytic factors by a murine tumor-producing cachexia in the host. *Cancer Res.*, **47**, 5919–5923.

BECK, S.A. & TISDALE, M.J. (1991). Lipid mobilizing factors specifically associated with cancer cachexia. *Br. J. Cancer*, **63**, 846–850.

BRENNEMAN, D.E. & SPECTOR, A.A. (1974). Utilization of ascites plasma very low density lipoprotein triglycerides by Ehrlich cells. *J. Lipid Res.*, **15**, 309–316.

COURANT, R. (1937). *Differential and Integral Calculus*. Vol. I, p. 343. Interscience Publishers, Inc.: New York.

DAUCHY, R.T. & SAUER, L.A. (1986). Preparation of 'tissue-isolated' rat tumors for perfusion: A new surgical technique that preserves continuous blood flow. *Lab. Animal Sci.*, **36**, 678–681.

FILLERUP, D.L., MIGLIORI, J.C. & MEAD, J.F. (1958). The uptake of lipoproteins by ascites tumor cells. The fatty acid-albumin complex. *J. Biol. Chem.*, **233**, 98–101.

FOLCH, J., LEES, M. & SLOANE-STANLEY, G.H. (1957). A simple method for the isolation and purification of total lipids from animal tissues. *J. Biol. Chem.*, **226**, 497–507.

FULLER, R.W. & DILLER, E.R. (1970). Diurnal variation of liver glycogen and plasma free fatty acids in rats fed ad libitum or a single daily meal. *Metabolism*, **19**, 226–229.

GULLINO, P.M. & GRANTHAM, F.H. (1961). Studies on the exchange of fluids between host and tumor. 1. A method for growing 'tissue-isolated' tumors in laboratory animals. *J. Natl Cancer Inst.*, **27**, 679–693.

GULLINO, P.M., GRANTHAM, F.H. & COURTNEY, A.H. (1967). Glucose consumption by transplanted tumors *in vivo*. *Cancer Res.*, **27**, 1031–1040.

HALPERIN, M.L., TAYLOR, W.M., CHEEMA-DHADLI, S., MORRIS, H.P. & FRITZ, I.B. (1975). Effects of fasting on the control of fatty-acid synthesis in hepatoma 7777 and host liver. *Eur. J. Biochem.*, **50**, 517–522.

IP, C., CARTER, C.A. & IP, M.M. (1985). Requirement for essential fatty acid for mammary tumorigenesis in the rat. *Cancer Res.*, **45**, 1997–2001.

KALLINOWSKI, F., VAUPEL, P., RUNKEL, S., FORTMEYER, H.P., BAESSLER, K.H., WAGNER, K., MUELLER-KLIESER, W. & WALENTA, S. (1988). Glucose uptake, lactate release, ketone body turnover, metabolic micromileau and pH distributions in human breast tumor xenografts in nude rats. *Cancer Res.*, **48**, 7264–7272.

KALLINOWSKI, F., SCHLENGER, K.H., KLOES, M., STOHRER, M. & VAUPEL, P. (1989). Tumor blood flow: The principal modulator of oxidative and glycolytic metabolism and of the metabolic micromileau of human tumor xenografts *in vivo*. *Cancer*, **44**, 266–272.

McDONALD-GIBSON, R.G. (1987). Quantitative measurements of arachidonic acid in tissues or fluids. In *Prostaglandins and Related Substances*, Benedetto, C., McDonald-Gibson, R.G., Nigam, S. & Slater, T.F. (eds) p. 259–268. IRL Press: Washington.

MERMIER, P. & BAKER, N. (1974). Flux of free fatty acids among host tissue, ascites fluid, and Ehrlich ascites carcinoma cells. *J. Lipid Res.*, **15**, 339–351.

SAUER, L.A., DAUCHY, R.T., NAGEL, W.O. & MORRIS, H.P. (1980). Mitochondrial malic enzymes. *J. Biol. Chem.*, **255**, 3844–3848.

SAUER, L.A., STAYMAN, J.W. & DAUCHY, R.T. (1982). Amino acid, glucose and lactic acid utilization *in vivo* by rat tumors. *Cancer Res.*, **42**, 4090–4097.

SAUER, L.A. & DAUCHY, R.T. (1983). Ketone body, glucose, lactic acid and amino acid utilization by tumors *in vivo* in fasted rats. *Cancer Res.*, **43**, 3497–3503.

SAUER, L.A., NAGEL, W.O., DAUCHY, R.T., MICELI, L.A. & AUSTIN, J. (1986). Stimulation of tumor growth in adult rats *in vivo* during an acute fast. *Cancer Res.*, **46**, 3469–3475.

SAUER, L.A. & DAUCHY, R.T. (1987). Stimulation of tumor growth in adult rats *in vivo* during acute streptozotocin-induced diabetes. *Cancer Res.*, **47**, 1756–1761.

SAUER, L.A. & DAUCHY, R.T. (1987*a*). Blood nutrient concentrations and tumor growth *in vivo* in rats; Relationships during the onset of an acute fast. *Cancer Res.*, **47**, 1065–1068.

SAUER, L.A. & DAUCHY, R.T. (1988). Identification of linoleic and arachidonic acids as the factors in hyperlipemic blood that increases $^3$H-thymidine incorporation in hepatoma 7288CTC perfused *in situ*. *Cancer Res.*, **48**, 3106–3111.

SAUER, L.A. & DAUCHY, R.T. (1992). The effect of omega-6 and omega-3 fatty acids on $^3$H-thymidine incorporation in hepatoma 7288CTC perfused *in situ*. *Br. J. Cancer*, **66**, 297–303.

SPECTOR, A.A. (1967). The importance of free fatty acids in tumor nutrition. *Cancer Res.*, **27**, 1580–1586.

SPECTOR, A.A. & BRENNEMAN, D.E. (1973). Role of free fatty acid and lipoproteins in the lipid nutrition of tumor cells. In *Tumor Lipids; Biochemistry and Metabolism*, Wood, R. (ed.) p. 1–13. American Oil Chemists Society: Champaign.

STEINAU, H.U., BASTERT, G., EICHHOLZ, H., FORTMEYER, H.P. & SCHMIDT-MATTHIESEN, H. (1981). Epigastric pouching technique: Human xenografts in rnu/rnu rats. In *Thymusaplastic Nude Mice and Rats in Clinical Oncology*, Bastert, G.B.A., Fortmeyer, H.P. & Schmidt-Matthiessen, H. (eds) p. 531–542. Gustav Fischer Verlag: Stuttgart.

WATSON, J.A. (1973). Regulation of cholesterol synthesis in HTC cells (minimal deviation hepatoma 7288CTC). In *Tumor Lipids: Biochemistry and Metabolism*, Wood, R. (ed.) p. 34–53. American Oil Chemists Society: Champaign.

WEBER, G., MORRIS, H.P., LOVE, W.C. & ASHMORE, J. (1961). Comparative biochemistry of hepatomas. II. Isotope studies of carbohydrate metabolism in Morris hepatoma 5123. *Cancer Res.*, **21**, 1406–1411.

WOOD, R. (1975). Hepatoma, host liver and normal rat liver as affected by diet. *Lipids*, **10**, 736–745.

ZOELLER, R.A. & WOOD, R. (1984). Analysis of the stearoyl-CoA desaturase system in the Morris hepatoma 7288C and 7288CTC. *Lipids*, **19**, 488–491.